# Prevalence of osteoporosis and predictors of low BMD in a cohort of HIV-1-infected patients in Rome: features of a population at high risk

**DOI:** 10.7448/IAS.17.4.19570

**Published:** 2014-11-02

**Authors:** Alessandro D'Avino, Annapia Lassandro, Silvia Lamonica, Benedetta Piccoli, Massimiliano Fabbiani, Annalisa Mondi, Roberta Gagliardini, Alberto Borghetti, Iuri Fanti, Federico Pallavicini, Roberto Cauda, Simona Di Giambenedetto

**Affiliations:** 1 Department of Clinical Infectious Diseases, Catholic University of Sacred Heart, Rome, Italy; 2 Department of Endocrinology, Catholic University of Sacred Heart, Rome, Italy

## Abstract

**Introduction:**

Ageing of HIV-infected patients led to an increasing rate of osteopenia and osteoporosis. The cause is multifactorial, including virus activity, drug toxicity and host factors. The aim of our analysis is to quantify this issue according to our department experience and to evaluate predictors of low BMD.

**Materials and Methods:**

HIV-1-infected patients, on stable HAART, were consecutively enrolled in this cross-sectional study and underwent DEXA. We analyzed the prevalence and evaluated predictors of low BMD in our population.

**Results:**

We collected data from 208 patients, 148 of whom were male, with 49 years median age (IQR 24.1–68.3). About 39% of patients were heterosexuals, 33.7 MSM and 12.5% were IDU, 40.4% were smokers. Caucasians were 93.3%, and 13.9% were co-infected with HCV virus. Around 6.7% of patients were on their first HAART regimen and all of them started TDF. Their median time of HAART exposure was 1.17 years (IQR 0.8–1.6). Conversely, median time of HAART exposure of multi-experienced patients was 8.5 years (IQR 3.1–12.0). We stratified DEXA results for patients on first-line regimen versus multi-experienced one. We found that 42.9% of patients on first-line HAART had low BMD of lumbar spine and 7.1% had osteoporosis. Regarding the multi-experienced group of patients, lumbar spine osteopenia was observed in 36.6% of patients and 15.5% of them had osteoporosis. Median age of patients with low BMD of lumbar spine was 45.6 (IQR 24.1–68.3) for patients on first-line regimen and 49.8 years for multi-experienced (IQR 44.2–54.0) regimen. We found similar data for BMD of hip, but no patients in the first group had hip osteoporosis. We also analyzed predictors of low BMD in our population. MSM patients showed a 3.4-fold higher risk to have osteoporosis of lumbar spine (OR 3.41, CI 1,105–9,269, p=0.03). As expected, we found that non-Caucasian patients had 13.5-fold higher risk to have osteoporosis of the hip (OR 13.52, CI 1.5–122.7, p=0.02). Exposure to HAART was also evaluated, but no predictors were found.

**Conclusions:**

Our data confirm how osteoporosis is highly prevalent and occurs earlier in HIV-infected patients. Antiretrovirals play a crucial role. In our experience loss of BMD can occur within a year of treatment, when almost half of our patients starting TDF had a low BMD. MSM patients have a higher risk to develop spine osteoporosis and non-Caucasian patients are more likely to have hip osteoporosis. We remark the importance of BMD assessment for HIV-infected patients especially during their first months of treatment.

